# Clinical application and prospect of immune checkpoint inhibitors for CAR-NK cell in tumor immunotherapy

**DOI:** 10.3389/fimmu.2022.1081546

**Published:** 2023-01-19

**Authors:** Kangdi Yang, Yuze Zhao, Guanqun Sun, Xu Zhang, Jinjin Cao, Mingcong Shao, Xijun Liang, Lina Wang

**Affiliations:** ^1^ Department of Traditional Chinese Medicine, Changhai Hospital, Naval Medical University, Shanghai, China; ^2^ School of Basic Medicine, Naval Medical University, Shanghai, China; ^3^ Clinical Cancer Institute, Center for Translational Medicine, Naval Medical University, Shanghai, China

**Keywords:** CAR-NK cell, tumor immunotherapy, immune checkpoint inhibitors, clinical, prospect

## Abstract

Chimeric antigen receptor (CAR) engineering of natural killer (NK) cells is an attractive research field in tumor immunotherapy. While CAR is genetically engineered to express certain molecules, it retains the intrinsic ability to recognize tumor cells through its own receptors. Additionally, NK cells do not depend on T cell receptors for cytotoxic killing. CAR-NK cells exhibit some differences to CAR-T cells in terms of more precise killing, numerous cell sources, and increased effectiveness in solid tumors. However, some problems still exist with CAR-NK cell therapy, such as cytotoxicity, low transfection efficiency, and storage issues. Immune checkpoints inhibit immune cells from performing their normal killing function, and the clinical application of immune checkpoint inhibitors for cancer treatment has become a key therapeutic strategy. The application of CAR-T cells and immune checkpoint inhibitors is being evaluated in numerous ongoing basic research and clinical studies. Immune checkpoints may affect the function of CAR-NK cell therapy. In this review, we describe the combination of existing CAR-NK cell technology with immune checkpoint therapy and discuss the research of CAR-NK cell technology and future clinical treatments. We also summarize the progress of clinical trials of CAR-NK cells and immune checkpoint therapy.

## 1 Introduction

Innate immunity, also known as non-specific immunity, is a natural immune defense function that was gradually formed during the long-term development and evolution of the body. As an innate immune cell type, natural killer (NK) cells actively participate in the first line of defense against invasion by pathogenic microorganisms (1).

In the past few years, research into chimeric antigen receptor (CAR)-modified NK cell therapy has increased. CAR-modified NK cell therapy, similar to CAR-T cell therapy, involves the expression of synthetic receptors by genetically modified immune cells; these immune cells are redirected to tumor surface antigens for tumor clearance through the cytotoxicity of immune cells (2, 3). Researchers have used NK cells from different sources with various modular CAR designs against a variety of target antigens (4–6). CAR-T cell therapy and CAR-NK cell therapy have many advantages, but they also have common disadvantages. Such as Immune exhaustion caused by immune checkpoints may be one of the common problems to be solved in clinical treatment (7).

Immune checkpoint molecules are immunosuppressive molecules that are expressed on immune cells and regulate the degree of immune activation (8). Upon activation, immune checkpoint molecules maintain the immune system within normal levels, so that the immune system is not overactivated, preventing autoimmunity. In the tumor microenvironment, tumor cells express immune checkpoint inhibitory ligands, thereby stimulating the downstream signaling pathway of immune cells, leading to immune exhaustion and providing a more suitable environment for tumor cell survival (9). Immune checkpoint immunotherapy is currently used to regulate the activity of T cells and NK cells to kill tumor cells through a series of pathways such as co-inhibition or co-stimulation signals (8, 10, 11).

Previous studies have explored the combination of CAR-T cells with immunotherapy targeting programmed cell death protein 1/programmed cell death 1 ligand 1 (PD-1/PD-L1) (12). However, studies on the combination of CAR-NK cell with immune checkpoints therapy are limited (13). This review describes the current research on the combination of CAR-NK and immune checkpoint therapies with the aim of providing insights for clinical and basic research for cancer treatment.

## 2 NK cells

### 2.1 Human NK cells

**Figure 1:**
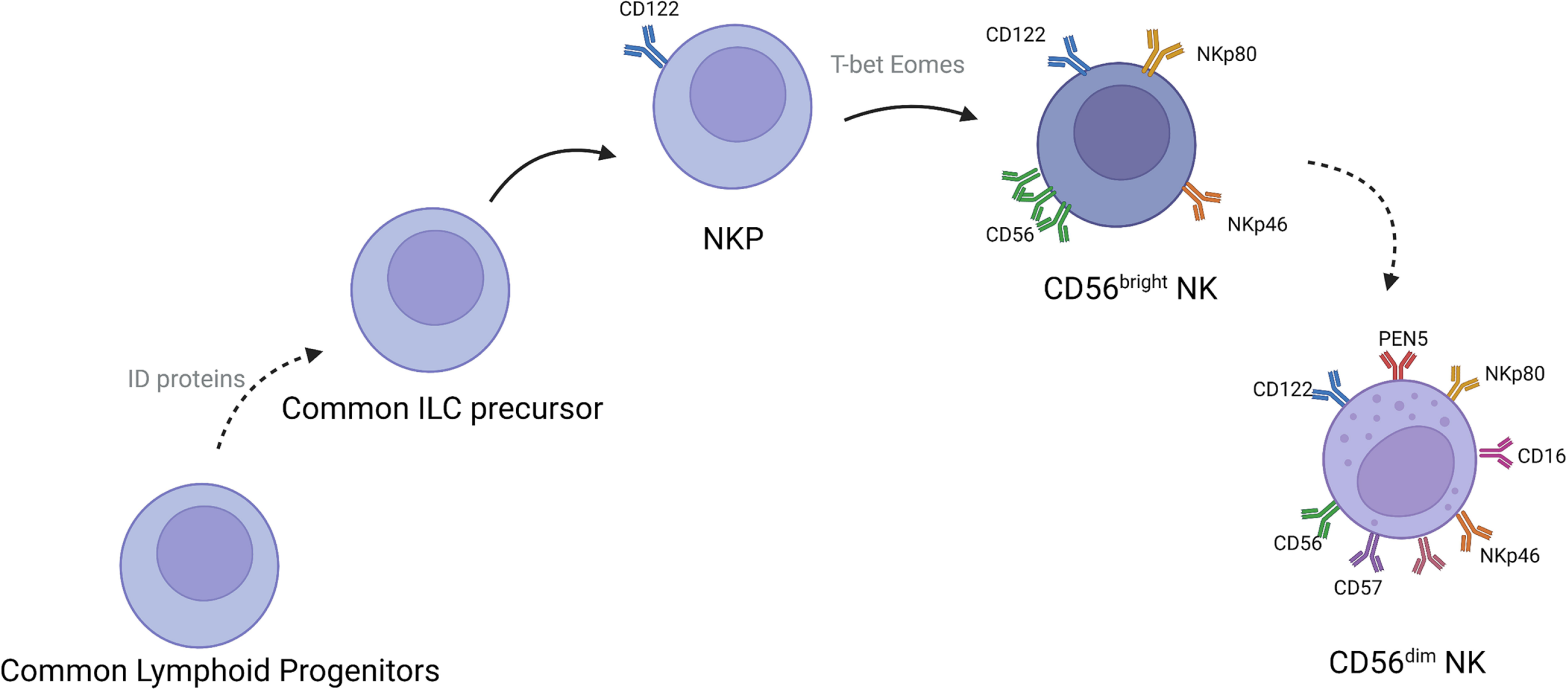
Development of NK cells in humans.

**Figure 2:**
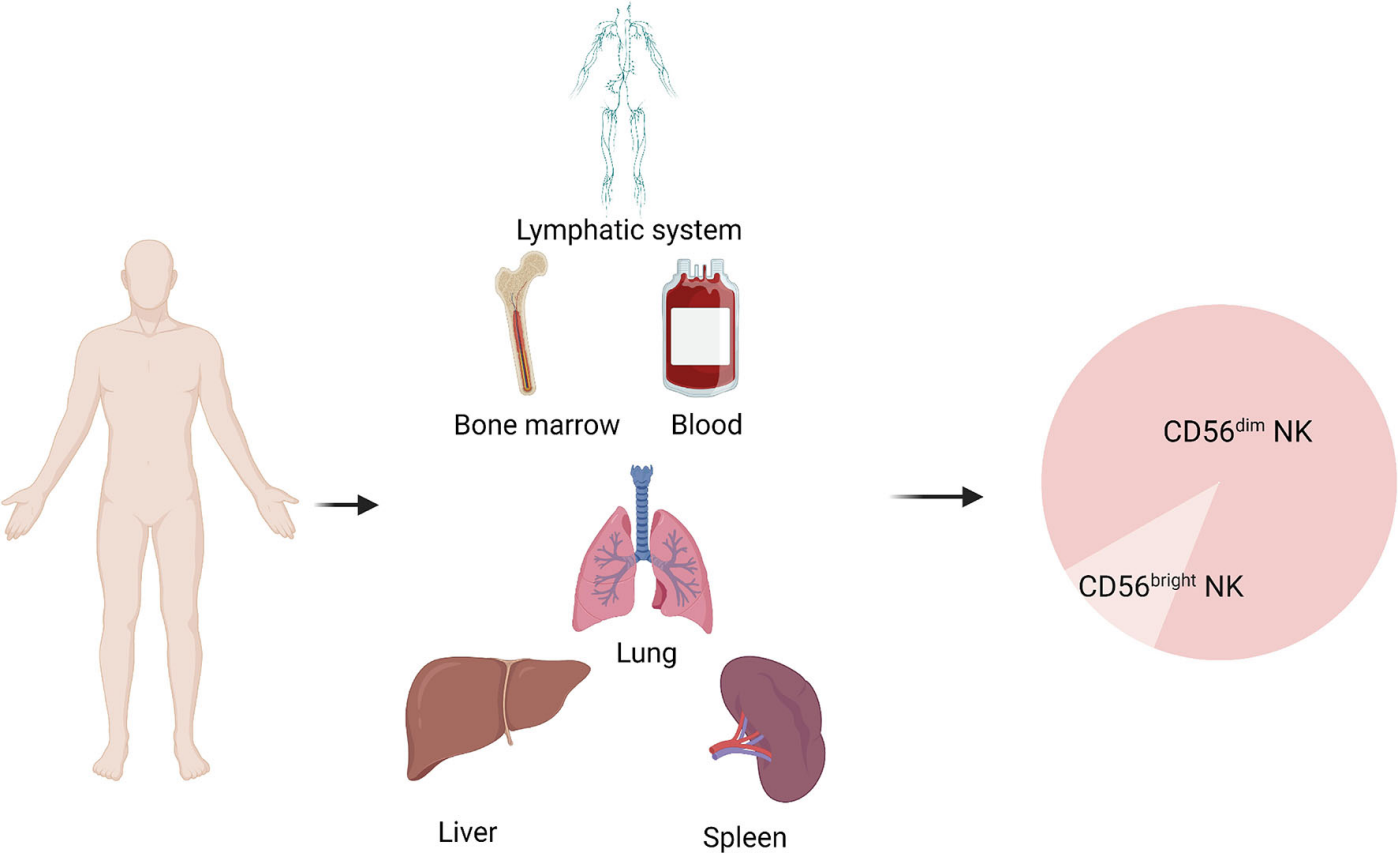
Distribution of NK cells in humans.

NK cells are mainly present in lymph nodes, bone marrow, peripheral blood, lungs, spleen, and liver (14). These cells develop from CD34+ hematopoietic progenitor cells. After developing into lymphoid progenitor cells, the cells gradually downregulate CD34 and upregulate CD56 and then develop into NK cells (**Figure 1**). CD56-expressing cells are divided into CD56^bright^ and CD56^dim^ subsets, defined on the basis of density of CD56 expression. More than 90% of NK cells subtype in the body is CD56^dim^ NK cells (15–17), which play an important roles in tumor immunotherapy (**Figure 2**). CD56^bright^ NK cells are immature NK cells and either function as progenitor cells of CD56^dim^ NK cells or as effector cells. Compare to CD56dim NK cells, CD56^bright^ NK cells exert less cytotoxic effects and secrete certain cytokines, growth factors, and chemokines to play immunomodulatory roles. CD56^dim^ NK cells exhibit weak cytokine secretion activity, but these cells show natural cytotoxicity and antibody-dependent cell-mediated cytotoxicity, with more lethality compared with CD56^bright^ cells (18–21). Their main target cells are tumor cells, virus-infected cells, and parasites, and these cells initiate and participate in the adaptive immune response. They also show good therapeutic effects in rheumatoid arthritis (22, 23).

### 2.2 NK cells target and kill tumor cells

Tumor development is caused by abnormal cell proliferation. Tumor progression involves metastasis from the primary site to other sites and invasion of vital organs and organ failure, resulting in patient death (24, 25). The body has various strategies to prevent tumor development through checks by the immune system (26). Therefore, the mechanisms by which NK cells find and kill tumor cells require elucidation to potentially identify new strategies to improve outcome of cancer patients.

**Figure 3:**
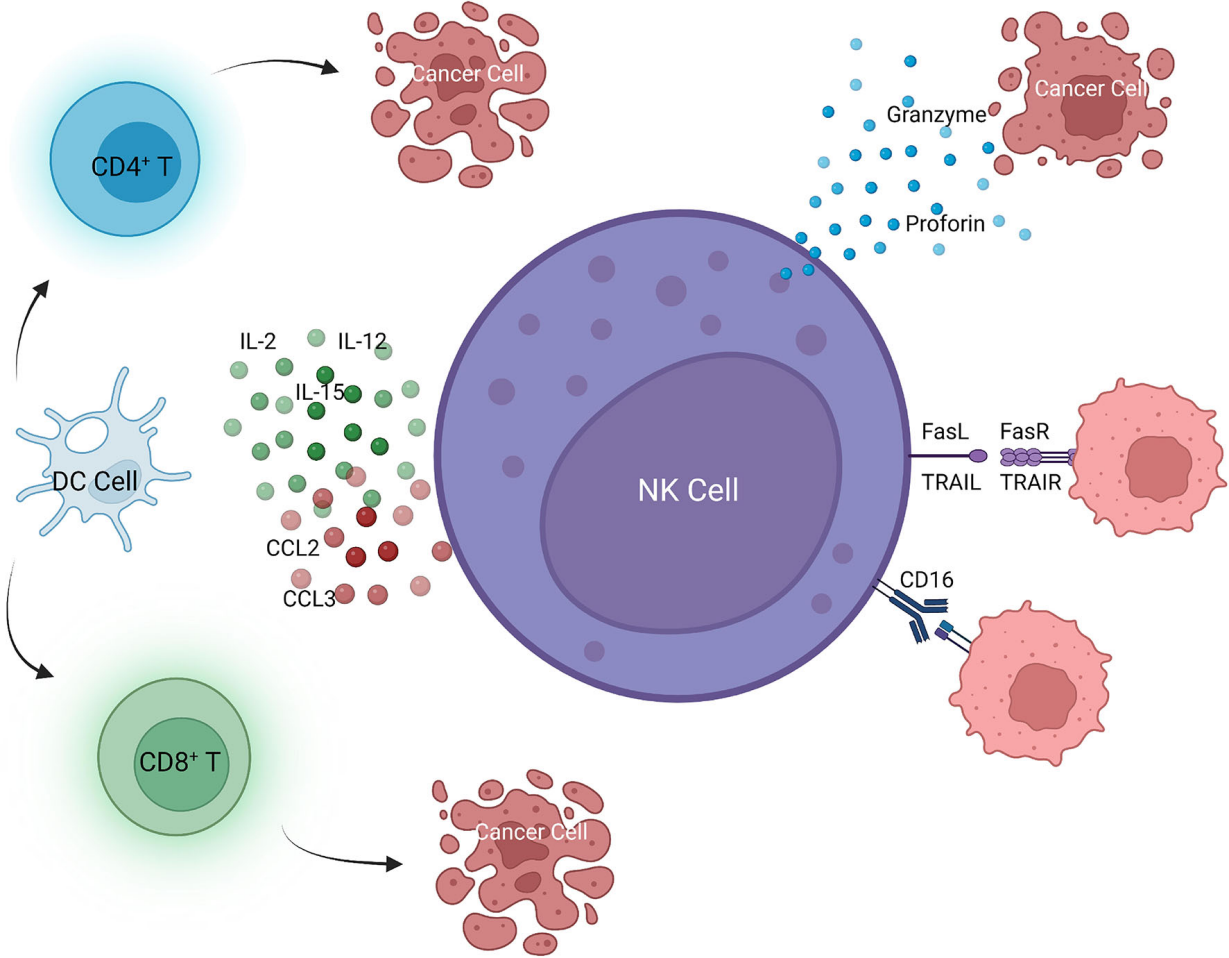
NK cell-mediated killing of tumor cells.

Tumor cells have reduced expression of major histocompatibility complex class I (MHC-I) early during tumor growth to avoid surveillance by the immune system. However, tumor cells with low MHC-I expression could stimulate NK cells, allowing NK cells to detect and kill tumor cells at an early stage (27, 28). NK cells eliminate tumor cells through four pathways. One is killing targeted tumor cells by releasing perforin and granzyme-containing cytoplasmic granules, leading to apoptosis of tumor cells. Granzymes are released into the intracellular space in a calcium-dependent manner (29, 30). Perforin in cytoplasmic granules induces cell membrane perforation, allowing granzyme to enter tumor cells, which leads to cell death receptor–mediated apoptosis (31). Second, NK cells can secret tumor necrosis factor (TNF) superfamily members, such as FasL and TNF-related apoptosis-inducing ligand (TRAIL), which can bind to their receptors to induce apoptosis of target cells (32, 33). The third pathway is to induce tumor cell apoptosis by limiting tumor angiogenesis and enhancing adaptive immunity by releasing effector molecules with anti-cancer properties, such as interferon-γ (IFN-γ) (29, 34). Stimulating cytokines, such as interleukin(IL)-2, IL-12, IL-15, and IL-18 and cytokines leading to IFN production, enhance the anti-tumor effect of NK cells (29, 35). NK cells also produce chemokines to recruit macrophages, dendritic cells, and T cells to cooperate in suppressing tumor growth (36, 37). Fourth, Fc receptor (CD16) is expressed and mediates the antibody-dependent cell-mediated cytotoxicity (ADCC) effect (38) (**Figure 3**).

### 2.3 Immune escape of tumor cells

Although NK cells play an important role in immune surveillance, tumor cells also could escape immune surveillance by NK cells through various mechanisms (39). Tumor cells can down regulate ligands recognized by NK cell receptors through metalloproteinase mediated cleavage and other mechanisms, leading to immune escape (40, 41). Second, during tumor development, tumor cells and factors in the tumor microenvironment release a variety of immunosuppressive factors to escape immune surveillance by NK cells (42). Third, NK cells are inhibited by immunosuppressive cells after immune escape of tumor cells (43).

## 3 Combined applications of CAR-NK cells and immune checkpoint inhibitors

Multiple studies have linked cancer and the immune system (44–46). Similar to organ transplantation, studies have shown that the immune system can recognize and respond to tumors. Therefore, research has focused on developing anti-tumor strategies by activating the immune system (47, 48). Tumor immunotherapy, including adoptive cell therapy and immune checkpoint therapy, are likely to revolutionize the treatment of malignant tumors (49). NK cell immunotherapy mainly includes adoptive NK cell therapy and NK cell–based ADCC functional antibody therapy. Adoptive NK cell therapy could exploit the intrinsic anti-tumor potentials of NK cells (50). Besides, NK cells can also be modified by gene editing (49, 51–53). However, these NK cell immunotherapies also have some limitations, such as life-threatening toxicity, insignificant efficacy in solid tumors, and poor durability [55]. Therefore, we propose combining CAR-immune cell therapy with other anti-tumor therapies to help improve the anti-tumor effect, inhibit toxicity, and improve the prognosis of patients (7, 12, 54–56).

### 3.1 CAR-NK cells

CAR-based gene modification of immune cells to express synthetic receptors redirects immune cells to tumor surface antigens for tumor clearance through immune cell cytotoxicity (57, 58). CAR molecules expressed on NK cells contain an extracellular domain, a transmembrane region, and one or more intracellular signaling domains (56, 59). The extracellular domain includes a signal peptide and an antigen-recognizing single-chain antibody fragment, which mainly recognizes tumor associated antigens (TAAs) on tumor cells. A hinge region connects this structure to the transmembrane region, which is also connected intracellularly to the intracellular domain containing the activation signal. The hinge assembly connects the ectodomain to the transmembrane domain. The transmembrane domain, a hydrophobic α-helix, crosses the membrane between the spacer and domain at end of the signal peptide. The inner domain (signaling domain) is relatively complex and is a functional component of CAR-immune cells that controls their activation, proliferation, and survival (60–62). Successful CAR design is achieved by a combination of careful design and functional testing. The inner domain of the CAR transmits costimulatory signals to immune cells in response to antigen recognition by the outer domain, enabling them to initiate cytotoxic functions (63). Similar to CAR-T cell therapy, several generations of CAR-NK cells have been developed. First-generation CAR-NK cells, similar to CAR-T cells, contain only CD3ζ signals. The CAR construct has been fine-tuned to induce a more potent anti-tumor response, increase antigen affinity, and prolong *in vivo* persistence using multiple genetic engineering technologies. Various costimulatory elements have been studied, such as second- and third-generation CAR-NK cells, which carry one or two additional costimulatory signals, respectively. Costimulatory molecules are derived from the immunoglobulin superfamily (CD28 and inducible costimulator), TNF receptor superfamily (4-1BB, OX40, CD40, and CD27), SLAM-related receptor family (2B4), and other domains including CD40L and Toll-like receptor (3, 56, 64–66). Compared with early CAR-NK constructs, which are mainly based on the costimulatory domain involved in T cell activation, NK cell–specific signal adapters become valuable due to their increasingly powerful functions (67, 68). In a preclinical study investigating CD19-directed CAR-NK cells, the addition of DAP10, a physiological adapter of NKG2D, resulted in enhanced anti-tumor potency compared with constructs using CD3ζ signals alone (4, 68, 69). Other studies have reported that the addition of DAP12 to prostate stem cell antigen–targeted CAR constructs and 2B4 to mesodermin-targeted CAR-NK cells amplifies anti-tumor effects (70, 71).

The structural design of the first three generations of CARs depended on the immune cell receptor domain, which has some limitations in cellular immunotherapy. Most current CAR constructs rely on the CD3ζ chain signaling domain, and strong activation signals are important to induce effective anti-tumor responses but they may also lead to rapid exhaustion of effector cells. Thus, a combination of costimulatory domains can be used to calibrate the desired immune cell response. Compared with 4-1BB-based CARs, CD28-based CARs have faster effector features and induce higher expression of IFN-γ, granzyme B, and TNF-α. However, this strong costimulatory signal also leads to activation-induced cell death. Conversely, 4-1BB-CD3ζ signaling preferentially induces memory-related genes and sustains anti-tumor activity. The reason may be that the 4-1BB domain ameliorates NK cell depletion induced by the CD28 domain (68, 70, 72, 73).

Fourth-generation constructs, termed armed CARs, are more effective and incorporate molecular payloads that confer additional features and functions to CAR-modified immune cells that are not present in any physiological immune cell receptor. This approach enables engineering of the CAR structure. Current clinical trials of CAR-NK cells are investigating second- and third-generation CAR-NK constructs that eliminate all circulating adoptive NK cells by inducing IL-15 expression to enhance caspase 9 activity to prevent adverse toxicity (13, 74, 75).

### 3.2 Advantages of CAR-NK cells

**Figure 4:**
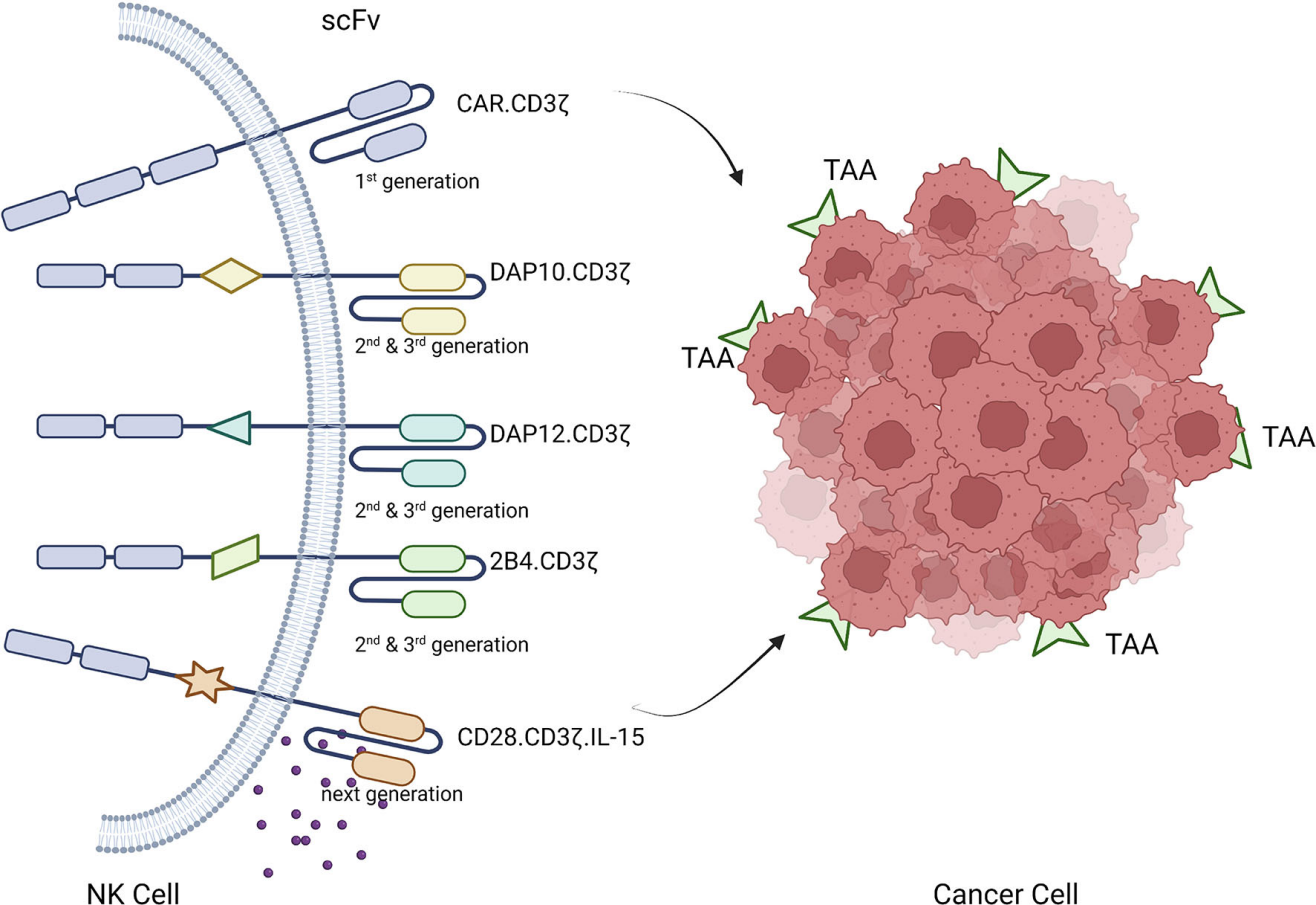
Development and working principle of CAR-NK cells.

CAR-NK cells have the potential to be applied in other medical fields. These cells may be safer than CAR-T cells because cytokines secreted by activated NK cells are safer and usually suppress the proinflammatory cytokines such as TNF-α, IL-1, and IL-6 released by CAR-T cells. Additionally, CAR-NK cells reduce the risk of GVHD because they are not restricted to MHC (51, 76). CAR-NK cells may also have various cytotoxic effects as they recognize and kill targets through engineered killing capabilities and natural cytotoxic receptors (77). Interestingly, Clinical trials CAR-NK cells can recognize and kill the residual tumor cells after long-term treatment. because CAR-NK cells contain CAR-dependent and CAR-independent target recognition and killing capabilities, the incidence of tumor escape in CAR-NK therapy is less (59, 78, 79). In addition, mature NK cells have a short lifespan in blood, which reduces the risk of cellular memory responses and cellular defects resulting from targeted/non-tumor effects (80). What is more, a large number of NK cell lines are available for CAR modification. Because of the low risks of alloreactivity and graft-versus-host disease (GVHD), allogeneic CAR-NK cells can be obtained from various sources including PB, UCB, iPSCs, hESCs, and NK-92 cells (13, 76, 81). Finally, the cost of CAR-NK cells is lower than that of CAR-T cells and thus CAR-NK may have greater market potential. The gradually improved technology makes it possible to store, thaw and reinfuse these cells, and can also carry out genetic engineering or genetic editing technology when needed (78) (**Figure 4**).

### 3.3 Disadvantages of CAR-NK cells

Despite the many advantages of NK cells, the application of CAR-NK cells has several challenges. Expansion of NK cells *in vitro* is the first limitation for CAR-NK cell immunotherapy. The number of NK cells obtained from a single donor is insufficient for treatment, which makes expansion and activation of NK cells critical (64, 82). Second, because the location of CAR binding epitope and its distance from CAR-NK cell surface affect its ability to bind antigen and activate CAR-NK cells, the current CAR used in NK cells has a structure that causes a first magnetic resistance, reducing the ability of these cells to bind antigens (83). Additionally, the production process of usually requires 2–3 weeks to culture NK cells and produce cytokines (IL-2 alone or in combination with IL-15 or an anti-CD3 monoclonal antibody) (13, 84). NK cells do not survive in the absence of cytokines. Therefore, exogenous cytokines must be provided to allow infused NK cells to survive and proliferate *in vivo* (64, 85). The source of NK cells is also an issue. Autologous NK cells need to be frozen and thawed. However, this reduces their anti-tumor effect and survival rate (86). Additionally, exogenous cytokines may have adverse effects such as systemic toxicity (70, 78, 87).

Similar to CAR-T cells, NK cells lack effective gene transfer strategies (88). Both viral and non-viral vectors have been used to genetically engineer CAR-NK cells (89). While the transduction efficiency of retroviral vectors is high, these vectors may cause insertional mutations, carcinogenesis, and other adverse effects (90). However, while lentiviral vectors have a low incidence of insertional mutations, their transfection efficiency in peripheral blood NK cells is as low as 20% (91). mRNA transfection is also considered to be a safe and practical transfection method for CAR-NK cells. In a xenograft tumor model, receptor expression exceeded 80% at 24h after electroporation of mRNA, and NK cells transfected with mRNA exerted marked cytotoxicity (92). Studies have recently shown that mRNA transfection avoids targeted non-tumor toxicity, a major limiting factor in the clinical application of CAR-modified cell immunotherapy (92, 93). However, the anti-tumor effect of CAR-NK cells transfected with mRNA by electroporation is temporary because the expression of CARs does not exceed 3 days.

## 4 CAR-NK cell and immune checkpoint therapies

**Figure 5:**
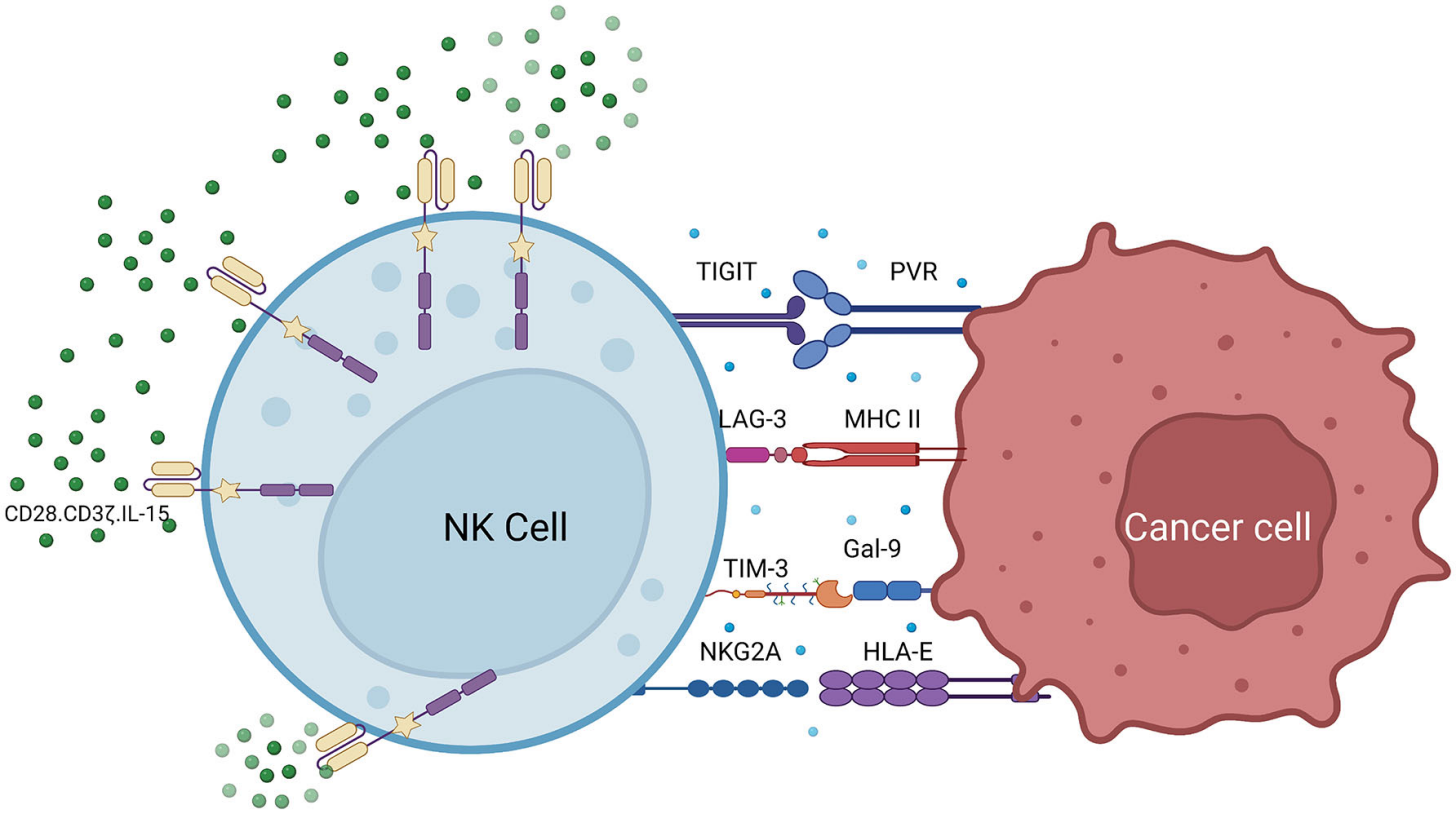
Combination of CAR-NK cells and immune checkpoint inhibitors.

CAR-NK cells have better targeting ability compared with other immune cells. In addition to recognizing tumor surface antigens through CARs, CAR-NK cells recognize tumor cells through various receptors such as natural cytotoxic receptors NKp46, NKp44, NKp30, NKG2D, and DNAM-1 (CD226) (13, 60, 94, 95). Despite the success of adoptive NK cell therapy, immune cell depletion remains a therapeutic barrier. To develop the next generation of CAR-NK cells, the negative regulator of NK cells may be a potential new design direction (96–98). T cell immune checkpoints affect CAR-T cell therapy. For example, PD-1 is an immune checkpoint receptor expressed on the T cell surface. It binds to PD-L1 expressed by target cells and sends an inactivating signal to T cells, thereby inhibiting the immune activity of T cells (12, 99, 100). However, many cancer cells express high levels of PD-L1, leading to cancer cell survival after T cell engagement. This can be overcome by targeted therapy. PD-1/PD-L1 axis inhibitors have been proven to achieve good clinical effects (101). However, PD-1 expression on NK cells is very low. Additionally, there are immune checkpoint receptors on NK cells that may regulate the functions of CAR-NK cells, such as T cell immunoreceptor with Ig and ITIM domains (TIGIT), NKG2A, lymphocyte activation gene-3 (LAG-3), and T cell immunoglobulin and mucin domain-containing protein 3 (TIM-3) (**Figure 5**) (95–97).

### 4.1 TIGIT

TIGIT, also known as WUCAM, Vstm3, and VSIG9, is a member of the poliovirus receptor (PVR)/Nectin family and immunoglobulin (Ig) superfamily (11, 102, 103). TIGIT is an inhibitory receptor (104) that inhibits T and NK cell activation (102). TIGIT is a transmembrane glycoprotein composed of three domains, the extracellular Ig variable domain, type I transmembrane domain, and short intracellular domain, and has an immune receptor tyrosine-based inhibition motif (ITIM) and immunoglobulin tyrosine tail-like phosphorylation motif (104, 105).

Previous studies have shown that TIGIT is expressed in all types of human NK cells (106). Poliovirus receptor *(*PVR, CD155), a physical ligand of TIGIT, which can bind TIGIT and activate immunosuppressive signal through the cytoplasmic ITIM domain of TIGIT (98). CD155 also plays an important role in both NK- and T cell-mediated immunity in humans and mice and is expressed on T cells, B cells, macrophages, and dendritic cells (105, 107, 108). CD155 is frequently overexpressed in human malignant tumors. As an immunomodulatory molecule, CD226 is a costimulatory molecule of T cells and NK cells, while TIGIT and CD96 are co inhibitory molecules, which can competitively bind CD155 (109). However, TIGIT has the highest affinity for CD155, and CD226 has the lowest affinity for CD155, as evidenced by direct radioligand binding analysis and competition experiments (110, 111). Therefore, the balance between the three competitively binding CD155 may play an important role in maintaining NK cells immune functions (112).

TIGIT interference has been shown to restore NK cell function and inhibit tumor growth in ovarian and breast cancer (113, 114). Notably, 58% sequence homology is found between human and mouse TIGIT; while the ITIM sequence in the cytoplasmic tail of TIGIT is the same in mice and humans (11, 107, 115). Similar to the inhibitory effect of human TIGIT, murine TIGIT also inhibits the cytotoxicity of mouse NK cells. Because of the cross-species specificity of the protein, human and mouse TIGIT have different binding properties. Human TIGIT binds more ligands to play an inhibitory role in immunity (104, 116).

### 4.2 NKG2A

NKG2A, an immunosuppressive receptor, is an attractive target for immunocheckpoint therapy. Approximately half of human peripheral blood NK cells express NKG2A (117). It is mainly expressed in CD56^bright^ NK cells and gradually decreases during NK cell maturation (118). NKG2A is a one-way type II integral membrane glycoprotein containing cytoplasmic, transmembrane, and extracellular lectin-like domains. NKG2A has two types of ITIM, which are mainly involved in immunosuppressive regulation (119–121).

The NKG2 protein is a C-type lectin that dimerizes with CD94 on the cell surface (122, 123). The non-classical MHC class I molecule human leukocyte antigen-E (HLA-E) is the main ligand of NKG2A/CD94, and its expression is approximately 25 times lower than that of classical MHC class I molecules. It is expressed in most normal tissues, and the interaction between NKG2A and HLA-E inhibits NK and T cell activation (97, 124, 125). Binding of NKG2A/CD94 receptors to peptide-presenting HLA-E leads to phosphorylation of ITIMs in NKG2A. Phosphorylated ITIMs are responsible for the recruitment and activation of intracellular phosphatases SHP-1 and SHP-2, thereby inhibiting the activation signals generated by activating receptors in NK cells (119, 126). HLA-E expression is generally increased in tumor cells (125), which provides NKG2A with more opportunities to inhibit NK cell activation. Similar to other immune checkpoint molecules, NKG2A is used by tumor cells for immune evasion. Therefore, disruption of the interaction between NKG2A and its ligands may enhance the anti-tumor immune response (127, 128). Previous studies have shown that blocking of inhibitory checkpoints in NK cells may also be effective for some metastatic carcinomas (129, 130). NKG2A is also involved in the pathological processes of immune-mediated diseases such as autoimmune diseases, inflammatory diseases, parasitic infections, and transplant rejection. These findings suggest that NKG2A is a novel therapeutic target for various immune-mediated diseases (131, 132).

### 4.3 LAG-3

Lymphocyte activation gene-3 (LAG-3), also known as CD223, is a 503 amino acid protein encoded by the LAG3 gene. LAG-3 is an immune checkpoint receptor protein localized in the cell membrane (133). The extracellular portion of the molecule consists of four immunoglobulin-like domains (D1–D4), which shows rigidity between D1 and D2 as well as D3 and D4 and relative flexibility between D1 and D2 as well as D3 and D4 (134). The human LAG3 gene is located on chromosome 12 (12p13), a similar location as the gene for CD4 (12p13.31). While LAG-3 and CD4 share only approximately 20% identical at the protein level, they have a highly homologous protein structure (135, 136). The cytoplasmic tail of LAG-3 has three key elements: a serine phosphorylation site, a KIEELE motif, and a glutamic proline dipeptide repeat. The KIEELE motif is highly conserved and may be involved in the transduction of the downstream inhibitory signal of LAG-3 because LAG-3 protein lacking this structure cannot exert an inhibitory effect on T cells (137). LAG-3 is selectively transcribed in activated T and NK cells. LAG-3 is mainly expressed in activated T, NK, B, and plasma cell dendritic cells. LAG-3 is expressed on NK cells, invariant NK T cells, Treg cells, and CD4+ and CD8+ subsets of T lymphocytes activated by antigens (138–141).

The role of LAG-3 in regulating NK cell functions is unclear, but similar to CD4, LAG-3 binds to major histocompatibility complex II (MHC-II) molecules. Compared with CD4, LAG-3 has a higher affinity for MHC-II (approximately 100 times) because LAG-3 enhances the interaction with MHC-II, and this interaction occurs through a ring structure composed of 30 amino acids in its D1 domain (138, 140, 142, 143). LAG-3 selectively binds to the stable antigen peptide–MHC-II molecular complex (pMHC-II). Therefore, LAG-3 preferentially inhibits the activation of CD4+ T cells with stable pMHC-II (143, 144). In NK cells, an increase in LAG-3 protein expression correlates with time post-infection and white pulp localization. One study suggested that upregulation of NK cells by LAG-3 causes the surrounding MHC-II+ cells to send inhibitory feedback signals, thereby terminating inhibition of T cells by NK cells (145). NK cells from LAG-3-deficient mice are defective in killing specific cancer cells (146). Considering of the effect of LAG-3 on the NK cell effector function, targeting LAG-3 may be useful in immunotherapy (147).

### 4.4 TIM-3

TIM-3 protein is a type I membrane protein also known as hepatitis A virus cell receptor 2 (HAVCR2), which is a negative regulator of anti-tumor immunity (148). It is a member of the TIM family that contain eight members, TIM1–TIM8. Among the protein family, TIM1, TIM3, and TIM4 are expressed in humans (149). TIM-3 was discovered in 2002 (150). TIM-3 includes three regions: the extracellular, transmembrane, and intracellular regions. The extracellular region consists of the N-terminal extracellular immunoglobulin variable (IgV) domain with an FG-CC loop and N-linked glycan, the mucin domain containing O-linked glycosylation sites, and the stalk domain containing N-linked glycan. The intracellular region consists of a cytoplasmic tail with five tyrosine residues (151, 152). TIM-3 is expressed on terminally differentiated CD4+ T cell subsets, such as Th1, Th17, and Tregs cells, and type 1 CD8+ T cells, but not on Th2 cells. It is also expressed on B cells, macrophages, dendritic cells, natural killer cells, mast cells, and monocytes (153, 154).

TIM-3 has been shown to inhibit tumor growth in various preclinical cancer models. The IgV domain contains binding sites for its ligands. Phosphatidylserine, carcinoembryonic antigen-associated cell adhesion molecule, and high mobility group protein 1 bind to the FG-CC loop, while Gal9 binds to N-linked glycans (151, 155). TIM-3 binds to its ligand galectin-9 to induce Th1 cell depletion (148, 156). The interaction of TIM-3 with its ligands also causes peripheral immune tolerance, and blocking TIM-3 eliminates the development of Th1 cell tolerance (148, 157). Although TIM-3 is a marker of T cell exhaustion (153), its expression is not associated with NK cell dysfunction in healthy donors. TIM-3 is also expressed in NK cells, the cytolytic activity of TIM-3^+^ NK cells from healthy humans is higher than that of TIM-3^-^ NK cells, and the TIM-3^+^ NK cells can kill K562 cells by releasing IFN-γ (158–160).

Cytokine stimulation increases the expression of TIM-3 on CD56^dim^ and CD56^bright^ NK cell subsets (161). TIM-3 expression on peripheral blood NK cells is increased in many cancer patients compared with healthy individuals. The expression of TIM-3 on NK cells increased with the development of disease stage. It has been reported that the survival rate of lung adenocarcinoma patients decreases with the increase of TIM-3^+^ NK cell percentage (155, 161, 162). Moreover, an study in esophageal cancer reported that tumor-infiltrating TIM-3+ NK cells showed a reduction in IFN-γ production and degranulation capacity compared with TIM-3+ counterparts (163). It has been reported that TIM-3 blockade can enhance the function of immune cells in multiple myeloma and melanoma (164, 165). Therefore, TIM-3 is expressed on fully functionally mature and/or activated NK cells and may function as an inhibitory receptor to inhibit NK cell functions similar to killer cell inhibitory receptor (KIR) and NKG2A.

## 5 Combination of CAR-NK cells therapy and immune checkpoint therapies for various tumors

Despite the remarkable success of adoptive NK cell therapy, immune cell depletion remains a barrier for therapeutic efficacy (166). To develop the next generation of CAR-NK cells, the identification of negative regulators of NK cell immune functions is required. Some checkpoint receptors, such as PD-1, LAG-3, TIM-3, TIGIT, and killer cell lectin-like receptor subfamily G member 1 (KLRG1), are upregulated in exhausted NK cells (96, 98). NKG2A is one of the most prominent inhibitory NK cell receptors, and its gene deletion is associated with increased NK cell cytotoxicity against tumors (117). Blocking TIGIT prevents NK cell depletion (102, 105, 107). Additionally, cytokine-inducible Src homology 2-containing (CIS) protein, which is an important cytokine checkpoint upstream of IL-15 signaling, is induced by the addition of cytokines, achieving enhanced metabolic fitness and effector functions in CAR-NK cells (70, 167). Other studies have revealed the positive effects of PD-1/PD-L1 and CTLA-4 blockade on NK cells (168).

CAR-NK therapy combined with immune checkpoint therapy showed better therapeutic effects compared with single therapy in clinical treatment (13). CAR-NK cell therapy has shown preliminary clinical significance. In addition to being effective against hematological and lymphoid tumors, NK cells have been used as an important treatment strategy for solid tumors (169, 170). For example, a phase I/IIa trial of CAR-NK cell therapy in 11 patients with relapsed/refractory *non-Hodgkin’s lymphoma* (NHL) or *chronic lymphocytic leukemia* (CLL) was recently reported (NCT00505245). Of the 11 patients in the trial, 8 patients (73%) were treated, and 7 patients had a complete response to the treatment with no evidence of disease at a median follow-up of 13.8 months. Most patients had a significant response within 30 days after receiving cell infusion, showing a progressive response, and the durability of the treatment was confirmed up to 1 year after infusion.

The safety of CAR-NK cell infusion has been shown by the absence of serious adverse events during patient treatment and follow-up (clinicaltrials.gov, accessed on January 1, 2020). While some patients in remission experienced disease relapse or required additional anti-cancer therapy, *cytokine release syndrome* (CRS), immune effector cell-associated neurotoxicity syndrome (ICANS), or GVHD of any grade has not been reported. Engineered hiPSC-derived allogeneic NK cells are expected to be a safe and effective off-the-shelf cell therapy drug (171). The function of transgenic NK cells is significantly enhanced and they have a significant killing ability for hematological and solid tumors. As of October 2022, 39 clinical trials of CAR-NK therapy have been registered, mainly in the United States, China, and European countries (clinicaltrials.gov). Most targets of CAR-NK therapy are in hematological tumors, but also in solid malignancies such as pancreatic, ovarian, and prostate cancers. Most clinical trials use allogeneic NK cells, mainly from healthy donors, or NK cell lines such as NK92 (**Table 1**).

**Table 1:** Clinical trial summary of CAR-NK cell therapies.

Cancer Type		Type of malignancy	NK Source	Title	Interventions	Statue	Phase	NCT Number
Neoplastic Hematologic Disorder	Lymphoma	B-cell Non Hodgkin Lymphoma b	cord blood	Clinical Study of Cord Blood derived CAR-NK Cells Targeting CD19 in the Treatment of Refractory/Relapsed B-cell NHL	Biological: anti CD19 CAR-NK	Recruiting	Phase 1	NCT05472558
		B-cell Non Hodgkin Lymphoma b	unpublished	Clinical Study of HLA Haploidentical CAR-NK Cells Targeting CD19 in the Treatment of Refractory/ Relapsed B-cell NHL	Biological: anti CD19 CAR-NK	Recruiting	Phase 1	NCT04887012
		NHL	unpublished	Anti-CD19 CAR NK Cell Therapy for R/R Non-Hodgkin Lymphoma.	Biological: anti CD19 CAR NK	Not yet recruiting	Early Phase 1	NCT04639739
		Refractory B-Cell Lymphoma	unpublished	Study of Anti-CD22 CAR NK Cells in Relapsed and Refractory B Cell Lymphoma	Biological: Anti CD22 CAR NK Cells	Unknown status	Early Phase 1	NCT03692767
		Refractory B-Cell Lymphoma	unpublished	Study of Anti-CD19 CAR NK Cells in Relapsed and Refractory B Cell Lymphoma	Biological: Anti CD19 CAR NK Cells	Unknown status	Early Phase 1	NCT03690310
		Non Hodgkin Lymphoma	unpublished	Anti-CD19 CAR-Engineered NK Cells in the Treatment of Relapsed/Refractory B-cell Malignancies	Biological: CAR NK-CD19 Cells	Recruiting	Phase 1	NCT05410041
		Refractory B-Cell Lymphoma	unpublished	Study of Anti-CD19/CD22 CAR NK Cells in Relapsed and Refractory B Cell Lymphoma	Biological: Anti CD19/CD22 CAR NK Cells	Unknown status	Early Phase 1	NCT03824964
		Non Hodgkin's Lymphoma	cord blood	Cord Blood Derived Anti-CD19 CAR-Engineered NK Cells for B Lymphoid Malignancies	Biological: Anti CD19/CD22 CAR NK Cells	Recruiting	Phase 1	NCT04796675
		•Lymphoma •Non_x005fHodgkin •Large B-cell Lymphoma •Mantle Cell Lymphoma •Indolent Lymphoma •Small Lymphocytic •Lymphoma Aggressive •Lymphoma •Large-cell Lymphoma	peripheral blood	NKX019, Intravenous Allogeneic Chimeric Antigen Receptor Natural Killer Cells (CAR NK), in Adults With B-cell Cancers	Biological: NKX019	Recruiting	Phase 1	NCT05020678
		•Follicular Lymphoma •Mantle Cell Lymphoma •Diffuse Large Cell Lymphoma	unpublished	PCAR-119 Bridge Immunotherapy Prior to Stem Cell Transplant in Treating Patients With CD19 Positive Leukemia and Lymphoma	Biological: anti CD19 CAR-NK cells	Unknown status	Phase 1 Phase 2	NCT02892695
		•Mantle Cell Lymphoma •Recurrent Diffuse Large B-Cell •Lymphoma Recurrent Follicular •Lymphoma Refractory B Cell Non-Hodgkin •Lymphoma Refractory Diffuse Large B-Cell •Lymphoma Refractory Follicular •Lymphoma	umbilical cord blood	CAR.CD19-CD28-zeta-2A iCasp9-IL15-Transduced Cord Blood NK Cells, High-Dose Chemotherapy, and Stem Cell Transplant in Treating Participants With B-cell Lymphoma	Procedure: Autologous Hematopoietic Stem Cell Transplantation •Drug: Carmustine •Drug: Cytarabine •Drug: Etoposide •Biological: Filgrastim •Drug: Melphalan •Biological: Rituximab •Biological: Umbilical Cord Blood-derived Natural Killer Cells	Withdrawn	Phase 1 Phase 2	NCT03579927
		•Acute Lymphocytic Leukemia •Non-hodgkin Lymphoma	cord blood	Umbilical & Cord Blood (CB) Derived CAR-Engineered NK Cells for B Lymphoid Malignancies	•Drug: Fludarabine •Drug: Cyclophosphamide •Drug: Mesna •Biological: iC9/ CAR.19/IL15- Transduced CB-NK Cells •Drug: AP1903	Active, not recruiting	Phase 1 Phase 2	NCT03056339
		•B-cell Lymphoma	unpublished	Natural Killer (NK) Cell Therapy for B-Cell Malignancies	•Drug: QN-019a •Drug: Rituximab •Drug: Cyclophosphamid •Drug: Fludarabine •Drug: VP-16	Recruiting	Phase 1	NCT05379647
		•B-Cell Lymphoma	unpublished	Phase I/II Study of CAR.70- Engineered IL15-transduced Cord Blood-derived NK Cells in Conjunction With Lymphodepleting Chemotherapy for the Management of Relapse/ Refractory Hematological Malignances	•Drug: Cyclophosphamide •Drug: CAR.70/IL15- transduced CB-NK cells •Drug: Fludarabine phosphate	Not yet recruiting	•Phase 1 •Phase 2	NCT05092451
		•Indolent Non Hodgkin Lymphoma •Aggressive Non-Hodgkin Lymphoma	unpublished	A Study of CNTY-101 in Participants With CD19-Positive B-Cell Malignancies	•Biological: CNTY-101 •Biological: IL-2 •Drug: Lymphodepleting Chemotherapy	Not yet recruiting	Phase 1	NCT05336409
	Leukemia	•Acute Myeloid Leukemia	unpublished	Study of Anti-CD33/CLL1 CAR NK in Acute Myeloid Leukemia	•Biological: NKG2D CAR-NK92 cells	Recruiting	Early Phase 1	NCT05215015
		•Leukemia, Myeloid, Acute	unpublished	Anti-CD33 CAR NK Cells in the Treatment of Relapsed/ Refractory Acute Myeloid Leukemia	•Biological: anti CD33 CAR NK cells •Drug: Fludarabine •Drug: Cytoxan	Recruiting	Phase 1	NCT05008575
		•Acute Lymphocytic Leukemia •Chronic Lymphocytic Leukemia	unpublished	Anti-CD19 CAR-Engineered NK Cells in the Treatment of Relapsed/Refractory B-cell Malignancies	•Biological: CAR NK-CD19 Cells	Recruiting	Phase 1	NCT05410041
		•Acute Lymphocytic Leukemia •Chronic Lymphocytic Leukemia	cord blood	Cord Blood Derived Anti-CD19 CAR-Engineered NK Cells for B Lymphoid Malignancies	•Drug: Fludarabine + Cyclophosphamide + CAR-NK-CD19 Cells	Recruiting	Phase 1	NCT04796675
		•Relapsed/ Refractory AML •AML, Adult	unpublished	NKX101, Intravenous Allogeneic CAR NK Cells, in Adults With AML or MDS	•Biological: NKX101 - CAR NK cell therapy	Recruiting	Phase 1	NCT04623944
		•B-cell Acute Lymphoblastic Leukemia •Waldenstrom Macroglobulinemia •Chronic Lymphocytic Leukemia	peripheral blood	NKX019, Intravenous Allogeneic Chimeric Antigen Receptor Natural Killer Cells (CAR NK), in Adults With B-cell Cancers	•Biological: NKX019	Recruiting	Phase 1	NCT05020678
		•Acute Lymphoblastic Leukemia	unpublished	Anti-CD19 CAR-Engineered NK Cells in the Treatment of Relapsed/Refractory Acute Lymphoblastic Leukemia	•Biological: CAR NK-CD19 Cells	Recruiting	Phase 1	NCT05563545
		•Acute Myelogenous Leukemia •Acute Myeloid Leukemia •Acute Myeloid Leukemia With Maturation •Acute Myeloid Leukemia Without Maturation •ANLL	NK-92 cell line	CAR-pNK Cell Immunotherapy for Relapsed/Refractory CD33+ AML	•Biological: anti CD33 CAR-NK cells	Unknown status	•Phase 1 •Phase 2	NCT02944162
		•Acute Lymphocytic Leukemia •Chronic Lymphocytic Leukemia •B-cell Prolymphocytic Leukemia	unpublished	PCAR-119 Bridge Immunotherapy Prior to Stem Cell Transplant in Treating Patients With CD19 Positive Leukemia and Lymphoma	•Biological: anti CD19 CAR-NK cells	Unknown status	•Phase 1 •Phase 2	NCT02892695
		•Acute Lymphoblastic Leukemia •Chronic Lymphoblastic Leukemia	cord blood	Universal Chimeric Antigen Receptor-modified AT19 Cells for CD19+ Relapsed/Refractory Hematological Malignancies	•Drug: Fludarabine + Cyclophosphamide + CAR-NK-CD19 Cells	Recruiting	Phase 1	NCT04796688
		•Acute Lymphocytic Leukemia •Chronic Lymphocytic Leukemia	unpublished	Umbilical & Cord Blood (CB) Derived CAR-Engineered NK Cells for B Lymphoid Malignancies	•Drug: Fludarabine •Drug: Cyclophosphamide •Drug: Mesna •Biological: iC9/ CAR.19/IL15- Transduced CB-NK Cells •Drug: AP1903	Active, not recruiting	•Phase 1 •Phase 2	NCT03056339
		•B-cell Acute Lymphoblastic Leukemia	unpublished	Natural Killer (NK) Cell Therapy for B-Cell Malignancies	•Drug: QN-019a •Drug: Rituximab •Drug: Cyclophosphamid •Drug: Fludarabine •Drug: VP-16	Recruiting	Phase 1	NCT05379647
		•Acute Myeloid Leukemia (AML)	cord blood	Phase I/II Study of CAR.70- Engineered IL15-transduced Cord Blood-derived NK Cells in Conjunction With Lymphodepleting Chemotherapy for the Management of Relapse/ Refractory Hematological Malignances	•Drug: Cyclophosphamide •Drug: CAR.70/IL15- transduced CB-NK cells •Drug: Fludarabine phosphate	Not yet recruiting	•Phase 1 •Phase 2	NCT05092451
	Myeloma	•Multiple Myeloma, Refractory	unpublished	Anti-BCMA CAR-NK Cell Therapy for the Relapsed or Refractory Multiple Myeloma	•Biological: Anti BCMA CAR-NK Cells •Drug: Fludarabine •Drug: Cytoxan	Recruiting	Early Phase 1	NCT05008536
		•Multiple Myeloma	unpublished	Clinical Research of Adoptive BCMA CAR-NK Cells on Relapse/Refractory MM	•Biological: BCMA CAR-NK 92 cells	Unknown status	•Phase 1 •Phase 2	NCT03940833
		•MDS •Refractory Myelodysplastic Syndromes	unpublished	NKX101, Intravenous Allogeneic CAR NK Cells, in Adults With AML or MDS	•Biological: NKX101 •CAR NK cell therapy	Recruiting	Phase 1	NCT04623944
		•Multiple Myeloma •Myeloma	peripheral blood	FT576 in Subjects With Multiple Myeloma	•Drug: FT576 (Allogenic CAR NK cells with BCMA expression) •Drug: Cyclophosphamide •Drug: Fludarabine •Drug: Daratumumab	Recruiting	Phase 1	NCT05182073
Solid Tumor		•Stage IV Ovarian Cancer •Testis Cancer Refractory •Endometrial Cancer Recurrent	peripheral blood	CLDN6-CAR-NK Cell Therapy for Advanced Solid Tumors	•Biological: Claudin6 targeting CAR-NK cells	Recruiting	•Phase 1 •Phase 2	NCT05410717
		•Refractory Metastatic Colorectal Cancer	NK-92 cell line	NKG2D CAR-NK Cell Therapy in Patients With Refractory Metastatic Colorectal Cancer	•Drug: NKG2D CAR-NK	Recruiting	Phase 1	NCT05528341
		•Relapsed/ Refractory Solid Tumors	NK-92 Cell line	NKG2D-CAR-NK92 Cells Immunotherapy for Solid Tumors	•Biological: NKG2D CAR-NK92 cells	Recruiting	Phase 1	NCT05528341
		•Advanced Solid Tumors	unpublished	Study of Anti-5T4 CAR-NK Cell Therapy in Advanced Solid Tumors	•Biological: Anti CAR-NK Cells	Recruiting	Early Phase 1	NCT05194709
		•Epithelial Ovarian Cancer	peripheral blood	Study of Anti-Mesothelin Car NK Cells in Epithelial Ovarian Cancer	•Biological: anti Mesothelin Car NK Cells	Unknown status	Early Phase 1	NCT03692637
		•Solid Tumours	peripheral blood	Pilot Study of NKG2D-Ligand Targeted CAR-NK Cells in Patients With Metastatic Solid Tumours	•Biological: CAR NK cells targeting NKG2D ligands	Unknown status	Phase 1	NCT03415100
		•SCLC, Extensive Stage	unpublished	Study of DLL3-CAR-NK Cells in the Treatment of Extensive Stage Small Cell Lung Cancer	•Biological: DLL3- CAR-NK cells	Recruiting	Phase 1	NCT05507593
		•Solid Tumor	unpublished	Clinical Research of ROBO1 Specific CAR-NK Cells on Patients With Solid Tumors	•Biological: ROBO1 CAR-NK cells	Unknown status	•Phase 1 •Phase 2	NCT03940820
		•Gastroesophageal Junction (GEJ) Cancers •Advanced HNSCC	unpublished	Immunotherapy Combination: Irradiated PD-L1 CAR-NK Cells Plus Pembrolizumab Plus N-803 for Subjects With Recurrent/ Metastatic Gastric or Head and Neck Cancer	•Drug: N-803 •Drug: Pembrolizumab •Biological: PD-L1 t haNK	Recruiting	Phase 2	NCT04847466
		•Pancreatic Cancer	unpublished	Clinical Research of ROBO1 Specific BiCAR-NK Cells on Patients With Pancreatic Cancer	•Biological: BiCAR NK cells (ROBO1 CAR-NK cells)	Unknown status	•Phase 1 •Phase 2	NCT03941457
		•Malignant Tumor	unpublished	Clinical Research of ROBO1 Specific BiCAR-NK/T Cells on Patients With Malignant Tumor	•Biological: BiCAR NK/T cells (ROBO1 CAR-NK/T cells)	Unknown status	•Phase 1 •Phase 2	NCT03931720
		•Metastatic Castration-resistant Prostate Cancer	unpublished	Study of Anti-PSMA CAR NK Cell (TABP EIC) in Metastatic Castration-Resistant Prostate Cancer	•Drug: TABP EIC •Biological: Cyclophosphamide •Biological: fludarabine	Recruiting	Early Phase 1	NCT03692663
Others		•Safety and Efficacy	unpublished	NKG2D CAR-NK Cell Therapy in Patients With Relapsed or Refractory Acute Myeloid Leukemia	•Biological: CAR-NK cells	Recruiting	Phase 1	NCT05247957
		•COVID-19	unpublished	A Phase I/II Study of Universal Off-the-shelf NKG2D-ACE2 CAR-NK Cells for Therapy of COVID-19	•Biological: NK cells,IL15-NK cells,NKG2D CAR NK cells,ACE2 CAR-NK cells,NKG2D-ACE2 CAR-NK cells	Recruiting	•Phase 1 •Phase 2	NCT04324996

*A* cytotoxic T-lymphocyte-associated protein 4 *(*CTLA4) inhibitor, ipilimumab, has been used to treat patients with advanced gastric cancer in a phase II clinical study (NCT01585987). Tremelimumab was evaluated in a phase II trial as a second-line treatment for patients with metastatic gastric adenocarcinoma. Pembrolizumab, a PD1 inhibitor, was approved by the FDA as a third-line treatment for patients with PD-L1-positive advanced gastric cancer. Nivolumab is a *Food and Drug Administration (*FDA)-approved drug as a third-line treatment for patients with advanced gastric cancer. Tebotelimab, another PD-1 inhibitor, blocks PD-1 and LAG-3 checkpoint molecules independently or synergistically. Additionally, durvalumab was used in a phase I B/II clinical trial in patients with advanced gastroesophageal cancer. In a phase III trial (NCT02625623), avelumab was used as third-line therapy. The TIGIT inhibitor tiragolumab is in a phase III trial and ociperlimab is in a phase II trial. Relatlimab, a LAG-3 inhibitor, has also entered clinical trials (**Table 2**).

**Table 2:** Clinical trials of CAR-NK cell and immune checkpoint inhibitor therapies.

Immune checkpoints	Drug	Number of CLINICAL TRIALS	In combination with CAR-NK	NCT Number	Conditions	Interventions
PD-1	Nivolumab Pembrolizumab Tislelizumab Camrelizumab Toripalimab	1271 3475 205 255 213	NO YES NO NO NO	NCT04847466	•Gastroesophageal Junction (GEJ) Cancers •Advanced HNSCC	•Drug: N-803 •Drug: Pembrolizumab •Biological: PD-L1 t_x005f haNK
PD-L1	Atezolizumab	570	NO			
TIGIT	Tiragolumab Vibostolimab Domvanalimab Ociperlimab Arcus	25 8 6 11 1	NO NO NO NO NO			
CTLA-4	Zalifrelimab BMS-986218 Quavonlimab BMS-986249 AGEN1181 BMS-986288 ADG-116 HBM-4003 ONC-392 YH-001 ADG-126 XTX101 JS007 BA3071	5 4 4 1 4 1 3 5 2 6 2 1 1 1	NO NO NO NO NO NO NO NO NO NO NO NO NO NO			
NKG2A	Monalizumab BMS-986315	9 1	NO NO			
TIM-3	TSR-022 LY3321367	4 1	NO NO			
LAG-3	GSK2831781 IMP321 BMS-986016	3 13 8	NO NO NO			
CD200R	Samalizumab	2	NO			
CD47	Magrolimab	20	NO			
B7-H3	Enoblituzumab MGA271	7 6	NO NO			

The combination of CAR-T cells and nivolumab (anti-PD-1 antibody) has been used in the treatment of relapsed or refractory classical Hodgkin’s lymphoma (CHL) (63). The PD-1/PD-L1 axis inhibits the cytotoxicity of CAR-T cells, thereby protecting tumor cells from being killed (56), which poses a challenge for CAR-T cell therapy. Therefore, the combination of CAR-NK cells therapy and immunocheckpoint therapy may become a new potential direction. CAR-NK will be developed into a safe, effective, and “off-the-shelf” cancer immunotherapy. In addition, immune checkpoint and CAR target can be designed together to optimize NK cell activation and cytotoxicity to overcome tumor suppression and escape.

## 6 Conclusion

In this review, we discussed CAR-NK therapy, preparation of CAR-NK cells, clinical progress, and the advantages and disadvantages of CAR-NK cells. Although CAR-NK cells have unique advantages, some challenges still exist, including the long time and high cost of CAR-NK cell preparation, biological toxicity, limited storage and transportation. The efficacy of CAR-NK cells in the treatment of solid tumors is limited. Regulatory challenges remain in terms of safety and clinical efficacy. We also propose the combination of CAR-NK cell and immune checkpoint therapies for future clinical applications. Ongoing research may resolve the challenges of CAR-NK cells and immune checkpoint therapies. Overcoming these issues will help provide new breakthroughs in the treatment of tumors by CAR modifications.

## Author contributions

KY and YZ were responsible for preparation of manuscript. GS, XZ, and MS were responsible for data collection. JC, KY, XL, and LW were responsible for supervision, reviewing,editing and funding acquisition. All authors contributed to the article and approved the submitted version.
